# Tobacco and alcohol use following first vs. consecutive cardiovascular events: a prospective longitudinal study

**DOI:** 10.3389/fcvm.2026.1786933

**Published:** 2026-03-25

**Authors:** Maria Teresa Pons-Cabrera, Karolina Szałata, Elsa Caballeria, Lourdes Navarro-Cortés, Clara Oliveras, Laura Bueno, Mercè Roqué, Carles Falces, Roger Borràs, Luis Pintor, Mercè Balcells, Hugo López-Pelayo

**Affiliations:** 1 Health and Addictions Research Group, IDIBAPS, Addictions Unit, Psychiatry and Psychology Service, ICN, Hospital Clinic Barcelona, Barcelona, Spain; 2 Medical University of Warsaw, Warsaw, Poland; 3 Psychiatry and Psychology Service, ICN, Hospital Clínic de Barcelona, Barcelona, Spain; 4 Cardiology Department, Clínic Cardiovascular Institute, Hospital Clinic-IDIBAPS, University of Barcelona, Barcelona, Spain; 5 Consultation and Liaison Psychiatry Section, Psychiatry and Psychology Service, ICN, Hospital Clínic de Barcelona, University of Barcelona, Barcelona, Spain

**Keywords:** alcohol consumption, cardiovascular risk factors, health behaviour, smoking cessation, tobacco smoking

## Abstract

**Aims:**

Examine tobacco and alcohol use patterns in a cohort of patients at their first (FE) or consecutive (CE) admission to a cardiology ward, and trajectories of use between groups over time.

**Methods:**

Tobacco and alcohol use was assessed at baseline and after one year on a prospective cohort of 249 patients admitted for CVD using the Timeline Followback method, and the Alcohol, Smoking and Substance Involvement Screening Test. Trajectories within and between groups were analysed using generalized multilevel mixed-effect models for repeated measures.

**Results and conclusions:**

Baseline prevalence of FE tobacco use was 29.5% and 15.4% in CE. The mean of weekly cigarettes was 35.8 for FE and 30.2 for CE (n.s.). The study found a significant effect of time on the cessation (*p* < 0.001) and the reduction (*p* = 0.018) of tobacco use. FE demonstrated a greater reduction in tobacco use prevalence (*p* = 0.003) and quantity (*p* < 0.001) –positive interaction between group and time. Baseline prevalence of alcohol use was 63.8% and 41.9% on FE and CE. Although there was a reduction in the prevalence and quantity of alcohol use in both groups, it remained relatively high. Although the occurrence of a cardiovascular event has a significant impact on the reduction of tobacco use, alcohol use appears more resistant to change post-event.

## 1 Introduction

Cardiovascular diseases (CVD) consistently remain one of the leading causes of illness and death, significantly contributing to healthcare expenditure (11% of the EU’s total healthcare expenditure) with 3 million deaths in Europe (1–3). However, a Cochrane systematic review and meta-analysis have shown that the adverse health effects of smoking can be partially reversed, even after the diagnosis of cardiovascular disease. For example, smoking cessation at diagnosis is associated with a reduction of about one third in the risk of recurrent cardiovascular (4).

Alcohol use represents another well-established risk factor for cardiovascular diseases, inflicting damage on the cardiovascular system, especially to the myocardium. In a report by the WHO, alcohol accounted for 7.2% of premature mortality globally, and contributed to 3.3% of CVD deaths in 2016, underscoring its substantial role as a modifiable factor impacting healthcare expenditures (5). Despite some research suggesting a positive impact of moderate alcohol use on certain cardiovascular diseases (6), more recent studies demonstrate that no amount of alcohol provides protection against CVD, and suggest a consistently escalating risk relationship between alcohol consumption and CVD, with the magnitude of risk increasing exponentially with higher intake (7).

The effectiveness of smoking cessation is influenced by several factors such as coronary disease severity, or the presence of psychiatric comorbidities (8, 9). Psychological factors, including awareness of associated risks, play a pivotal role in achieving tobacco abstinence. Despite a relatively low rate of unassisted smoking cessation, brief advice from a health professional can increase the rate by an additional 1%–3%, with more intensive interventions showing greater impact (10). Similarly, brief interventions can contribute to reduce alcohol intake among hazardous and harmful users (11). Disease detection has been shown to influence tobacco and alcohol use behaviours. A study by Kwon et al. indicated that inclusion in health screening programs, and the diagnosis of hyperlipidaemia, promote positive changes in smoking habits (12). Neutel and Campbell reported an association between hypertension diagnosis and smoking cessation, though no significant changes in risky alcohol use were observed (13). Indeed, other studies revealed that individuals who received the recommendation to cease smoking due to a smoker's related illness risks or were concerned about tobacco health related harms, are more likely to cease, especially with physician support (14–16). However, the impact of the first hospital admission or diagnosis of myocardial or vascular disease—collectively referred to as CVD in this paper—on tobacco and alcohol cessation remains inadequately explored.

The exploratory study aims to 1) assess the prevalence of tobacco and alcohol use, and tobacco and alcohol use patterns, in a single-centre cohort of patients at their first or consecutive admissions to a Cardiology ward, and 2) examine the trajectories and differences in tobacco and alcohol use between both groups over one year after the index hospitalization.

## 2 Methods

### 2.1 Study design

We analysed data from a prospective cohort of patients recruited between May and December 2021 during their hospitalization in the Cardiology ward of a high-complexity University community hospital in Barcelona due to a cardiovascular event requiring hospitalisation. The recruitment process was carried out by [MP] (psychiatrist), [EC] (psychologist), and [LN] (psychiatry trainee).

The study follows a prospective design, incorporating data during the index hospitalization, and a subsequent follow-up telephonic visit after one year, and represents a secondary analysis of a larger sample of patients recruited during their hospitalization in different medical wards (cardiology, but also pneumology, gastroenterology, and internal medicine) during May 2021 and February 2023. The study protocol received approval by the Ethics Committee of the [HCB/2020/1126] and the study was conducted in accordance with Good Clinical Practice guidelines and ethical principles stated in the Declaration of Helsinki 1964, as revised at the 64th World Medical Association General Assembly in Fortaleza, Brazil, October 2013.

As this is an exploratory analysis, the study aims to identify potential patterns and generate hypotheses regarding the trajectories of patients with cardiovascular events.

### 2.2 Participants

Inclusion criteria were being 18 years or older, willingness and availability to participate during hospitalization in one of the four medical wards during the recruitment period, and admission duration of less than 15 days (to mitigate memory bias). Exclusion criteria included the presence of a communication barrier (e.g., language) that could interfere the interview, cognitive impairment affecting study comprehension (observed during the conversation or by a diagnosed dementia), death during admission, residents outside Catalonia (to ensure the feasibility of follow-up data collection), or poor general health status that hindered effective participation.

Recruitment was halted temporarily during periods of COVID-19 pandemic surges, that required hospital restructuring, as well as times when service activity was reorganized due to holidays and non-working days.

### 2.3 Clinical assessment

All participants were assessed by a psychiatrist or psychologist at baseline during their hospitalization (T1) and follow-up (T2), one year later in a telephonic interview.

At baseline (T1) we obtained data regarding substance use, clinical data, demographic, and socio-economic data with the following instruments:

- Timeline Followback method (TLFB): to assess tobacco and alcohol use patterns we measured both frequency and quantity of use. For tobacco, patients reported the number of cigarettes consumed per week, and for alcohol, the Standard Drink Units (17) per week for the previous 3 months.
- The Alcohol, Smoking, and Substance Involvement Screening Test (ASSIST) (18): the substance use or potential substance use disorders were evaluated by the Spanish version of With ASSIST scores substance use, and associated risks fall on a continuum ranging from “lower risk” (occasional or non-problematic use), and “moderate risk” (more regular use), to “high risk” (frequent high-risk use or dependence).
- Biomarkers in urine test (Ethylglucuronide for alcohol and cotinine for tobacco): For patients who denied the use of tobacco or alcohol and were within the detection window period (72 h) for both substances in urine, a urinary analysis was conducted.
- Charlson Comorbidity Index (CCI): Clinical data on comorbidities was weighted using medical records and completing the to categorize medical comorbidities of CCI and the complexity of the case. Stepwise increases in mortality are seen with stepwise increases in CCI (19).
- Spanish version of the Mini International Neuropsychiatric Interview, version 5.0.0 (MINI) (20): to asess the presence of a psychiatric comorbidities.
- Adhoc questionniare for socio-demographic characteristics: gender, age, occupational status, education, and marital status

After one year (T2), patients TLFB and ASSIST self-reported were administered by a second telephonic interview. The mortality after one year was checked in medical records.

### 2.4 Statistical analysis

The study cohort was categorized analytically into two groups: patients admitted for a first episode of CVD labelled as First Episode (FE), and those hospitalized due to Consecutive Episodes (CE). Outcome evaluations were conducted at two time points – during the index hospitalization (T1) and one year later (T2). Categorical variables in descriptive analyses were expressed as total number with corresponding percentages and inter-group comparisons were conducted using either Pearson's Chi-squared test or Fisher's exact test as appropriate. Continuous variables were presented as mean ± standard deviation, and group mean comparisons utilized either the Student's t test or Wilcoxon rank sum test, as appropriate.

Trajectories within and between groups over the one-year longitudinal follow-up were analysed using generalized multilevel mixed-effect models for repeated measures, incorporating time per group as longitudinal interaction fixed effects. In all models, participant identification was treated as a random effect to account for within-participant correlations. For the model fitting, we used the Lme4 R package (v. 1.1.31). Residual plots were used to perform model validation. The analyses were adjusted by sex, age, severity of medical comorbidity (using CCI), the presence of a psychiatric comorbidity (using the MINI neuropsychiatric interview), and the ASSIST score for the outcome substance (degree of substance-related risk).

Statistical comparisons were executed using emmeans library (v. 1.8.2). All analyses were addressed considering a two-tailed type 1 error of 5%. For multiple *post hoc* comparisons, *P*-values were adjusted using the Tukey method. All statistical analyses were conducted using R (v. 4.2.2).

## 3 Results

### 3.1 Demographic and clinical characteristics in FE and CE

**Figure 1:**
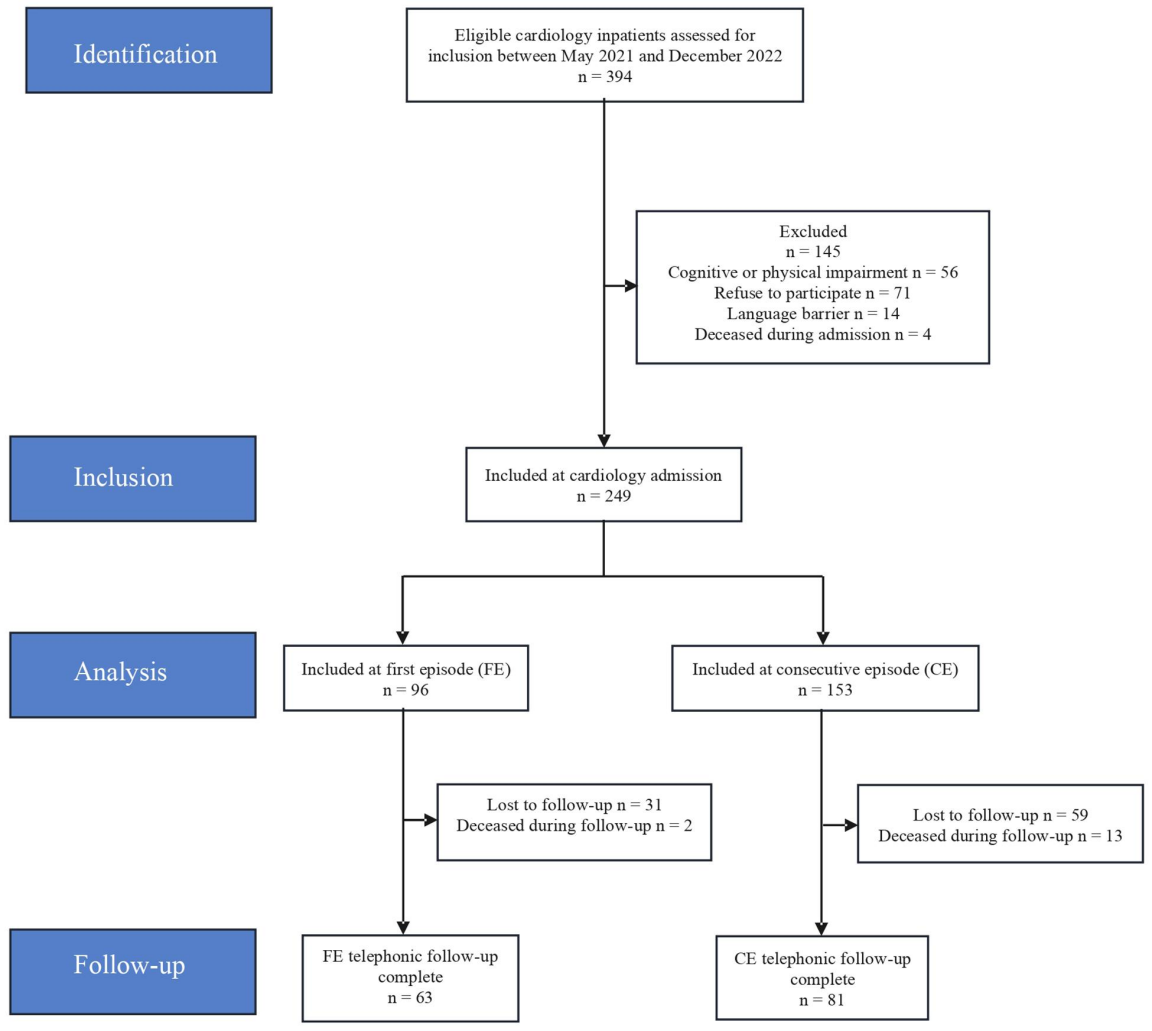
STROBE flow diagram.

Over the active recruitment period, 394 eligible patients were admitted, after selection criteria, a final sample of 249 patients during their stay in the Cardiology ward (Figure 1) were recruited, comprising 96 hospitalized for their first cardiovascular episode (FE, 38.4%) and 153 with consecutive admissions (CE, 61.2%). Groups differed significantly in several baseline characteristics. See Table 1 for demographic and clinical characteristics and Figure 1 for the recruitment flow diagram. The FE group was younger (mean age 59.8 ± 13.2 vs 64.6 ± 13.1 years, *p* = 0.005) and had significantly higher employment rates (50.5% vs 30.3%, *p* = 0.001). Comorbidity burden, as measured by the Charlson Comorbidity Index, was lower in the FE group, with 80.2% classified as mild compared to 54.9% in the CE group (*p* < 0.001). Groups did not differ in sex distribution (19.8% vs 27.5% female, *p* = 0.171) or marital status (*p* = 0.266).

**Table 1:** Comparisons across groups in demographic and clinical variables.

Baseline characteristics	FE *N* = 96 (38.4%)	CE *N* = 153 (61.2%)		
	Mean (SD)	Mean (SD)	t	*P*-value
Age	59.8 (13.2)	64.6 (13.1)	2.81	0.005*
LOS (days)	7.2 (4.9)	9.91 (8.5)	2.86	0.005*
	Prevalence % (*n*)	Prevalence % (*n*)	*χ*2	*P*-value
Gender (%female)	19.8 (19)	27.5 (42)	1.87	0.171
Employment status (% working/studying)	50.5 (48)	30.3 (46)	10.18	0.001*
Marital status (% w partner)	65.3 (62)	58.2 (89)	1.23	0.266
Death at FU	2.1 (2)	8.5 (13)	4.33	0.037*
Psy comorbidity	31.3 (30)	25.5 (39)	0.97	0.32
CCI index			19.99	<0.001*
WO comorbidity	10.4 (10)	12.4 (19)		
Mild	80.2 (77)	54.9 (84)		
Moderate	8.3 (8)	24.8 (38)		
Severe	1 (1)	7.8 (12)		
ASSIST TOB			10.09	0.006*
Lower risk	45. 3 (43)	65.4 (100)		
Moderate risk	46.3 (44)	30.7 (47)		
High risk	8.4 (8)	3.9 (6)		
ASSIST ALC			2.12	0.34
Lower risk	89.5 (85)	89.5 (137)		
Moderate risk	10.5 (10)	8.5 (13)		
High risk	0 (0)	2 (3)		

FE, patients hospitalized after a First Episode of cardiovascular disease; CE, patients hospitalized after a Consecutive Episode of cardiovascular disease; LOS, length of stay; SD, standard deviation; w, with; FU, follow-up; Psy comorbidity, presence of a psychiatric comorbidity; WO, without; TOB, tobacco; ALC, alcohol.

*Statistical significance (*p* < 0.05).

### 3.2 Tobacco use patterns in FE vs. CE

A significant main effect of time (over a one-year follow-up period) was observed for all variables of tobacco use. A significant interaction between group and time was found for tobacco use outcomes (see Table 2).

**Table 2:** Main effects of group, time, and interaction group by time in substance use patterns.

	FE		CE		Effects		
Substance use	Baseline	1 year follow-up	Baseline	1 year follow-up	Time	Group	Group*time
	Adjusted prevalence (95% CI)	Adjusted prevalence (95% CI)	Adjusted prevalence (95% CI)	Adjusted prevalence (95% CI)	*P*-value	*P*-value	*P*-value
Tobacco use	29.5 (14.9–50.0)	0.4 (0.01–1.8)	15.4 (7.7–28.3)	4.5 (1.5–13.1)	<0.00*	0.8	0.003*
Alcohol use	63.8 (45.8–78.6)	35.4 (18.5–56.9)	41.9 (28.0–57.2)	24.4 (12.6–42.0)	0.003*	0.054	0.54
	Adjusted mean (95% CI)	Adjusted mean (95% CI)	Adjusted mean (95% CI)	Adjusted mean (95% CI)	*P*-value	*P*-value	*P*-value
Cigs/w	35.8 (26.35–45.34)	0 (0–10.65)	30.2 (22.78–37.72)	19.5 (10.44–28.54)	0.018*	0.17	<0.001*
SDU/w	5.9 (3.49–8.33)	3.4 (0.46–6.3)	6.9 (5.04–8.86)	3.1 (0.72–5.5)	0.008*	0.75	0.56

cigs/w, number of cigarettes per week for the previous three months; SDU/w, number of standard drink units per week for the previous three months; FE, patients hospitalized after a First Episode of cardiovascular disease; CE, patients hospitalized after a Consecutive Episode of cardiovascular disease; CI, confidence interval.

This analysis was controlled by sex, age, marital status, the presence of a psychiatric comorbidity according to MINI, the Charlson Comorbidity Index, and the ASSIST scores for tobacco (for tobacco use and the number of cigarettes per week), and the ASSIST scores for alcohol (for alcohol use and number of SDU per week).

*Statistical significance (*p* < 0.05).

**Figure 2:**
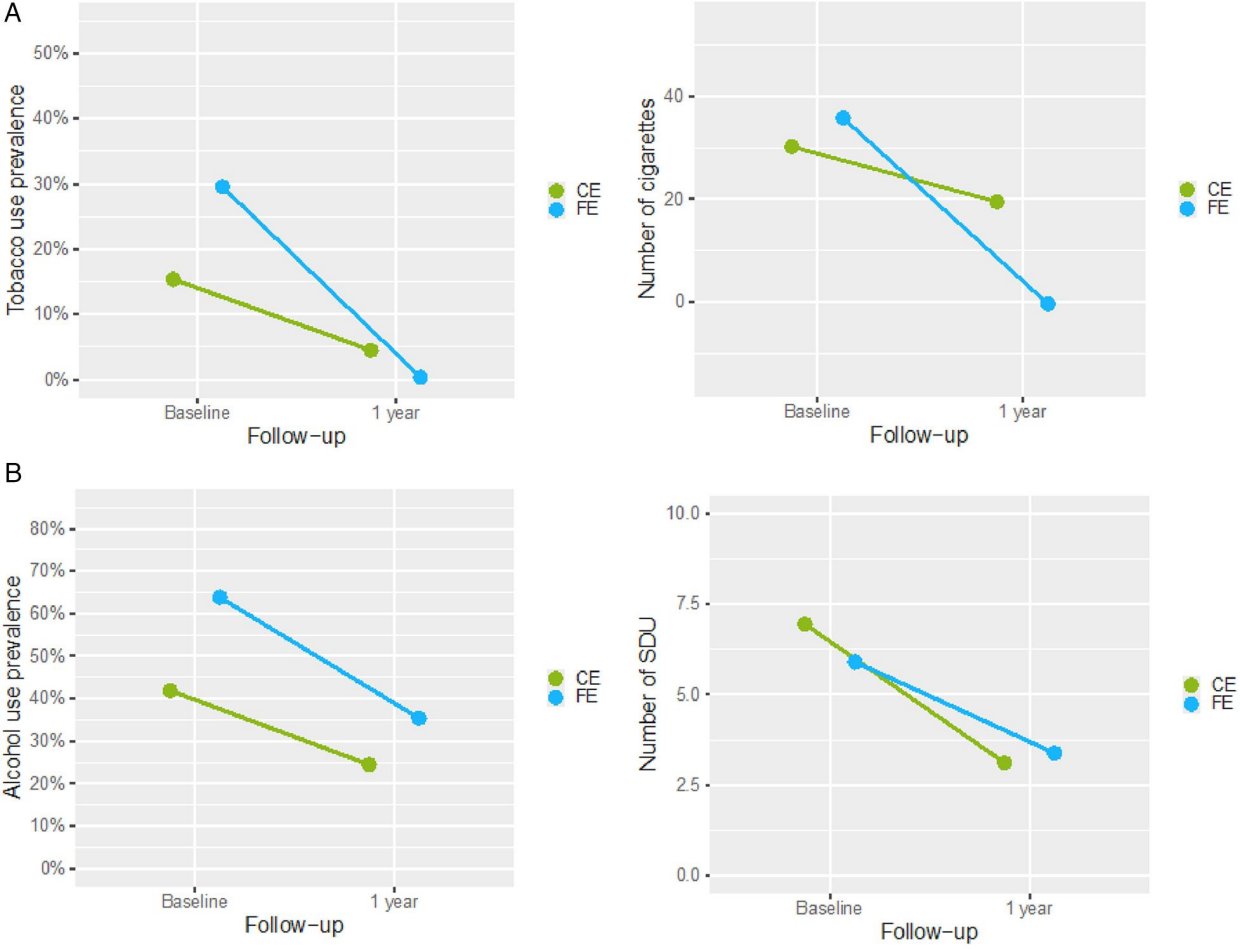
Changes in adjusted prevalence and consumption of tobacco and alcohol in FE and CE over time. FE, patients hospitalized after a First Episode of cardiovascular disease; CE, patients hospitalized after a Consecutive Episode of cardiovascular disease; SDU, standard drink units. **(A)** Tobacco use: prevalence and number of cigarettes per week. **(B)** Alcohol use: prevalence and number of SDU per week.

The adjusted baseline prevalence of tobacco use among FE patients was 29.5%, diminishing to 0.4% after one year. In the CE group, baseline adjusted prevalence of tobacco use was 15.4%, decreasing to 4.5% after one year. Significant time effects (*p* < 0.001), and group by time interactions were observed (*p* = 0.003.) The interaction effect revealed differing trajectories between FE and CE (Figure 2A).

A significant decrease over time was observed in the adjusted weekly tobacco amount (*p* = 0.018). In the FE group, tobacco amount decreased from an average of 35.8 cigarettes to total cessation in nearly all individuals, as suggested by the adjusted prevalence data. In the CE group, consumption decreased from 30.2 at baseline to 19.5 cigarettes weekly at follow-up. The significant interaction between group and time showed a non-uniform reduction between FE and CE groups (Figure 2A).

### 3.3 Alcohol use patterns in FE vs. CE

A significant main effect of time (over a one-year follow-up period) was observed for all variables of alcohol use. A significant interaction between group and time was not found for alcohol use, as measured by adjusted prevalence and number of SDU used weekly over the prior three months (see Table 2).

The adjusted prevalence of alcohol use was 63.8% among FE patients at baseline, which was reduced to 35.4% after one year. In CE patients, the adjusted prevalence decreased from 41.9% to 24.4%. Although a significant main effect of time was found (*p* = 0.003), no significant effects for group or interaction between group and time were observed (Figure 2B).

Regarding the weekly amounts of alcohol used, a significant main effect of time was found as well (*p* = 0.008), reflecting a reduction in both groups after one year. In FE, the mean weekly SDUs decreased from 5.9 to 3.4. In CE, SDUs decreased from 6.9 to 3.1. The absence of significant main effects group by time interaction indicates that the trajectories of reduction in weekly alcohol quantities did not differ significantly between the FE and CE groups (Figure 2B*)*.

## 4 Discussion

This study explores the trajectories of tobacco and alcohol use among 249 patients admitted due to cardiovascular diseases, distinguishing between first episode (FE) consecutive episodes (CE). At baseline, both groups exhibited higher adjusted prevalence of tobacco (29.5% FE, 15.4% CE) and alcohol use (63.8% FE, 41.9% CE). After one year, most patients had quit smoking, though alcohol use remained relatively high (24.4-35.4%). A significant interaction between group and time for tobacco was observed, with FE patients being more likely to quit smoking. Similarly, this interaction was apparent in the reduction of tobacco amount of use, indicating a more pronounced decrease in consumption among those admitted for their first cardiovascular event. However, no similar interaction was found for alcohol use outcome variables.

These findings suggest that hospitalization for a cardiovascular event can affect particularly the decision to cease smoking during the next year, especially after the first admission. This aligns with previous research, suggesting that hospitalizations, may serve as a “teachable moment” and catalyse changes in substance use patterns (21). Our findings on the lower success rate of smoking cessation following consecutive admissions for cardiovascular events are consistent with recent studies indicating that continued smoking is often predicted by a history of myocardial infarction (22).

Moreover, WHO directions advocate for the implementation of integrated brief interventions, recognized effective to address and change the exposure to all behavioural risk factors for non-communicable diseases, including CVD (23). The potential impact of such approaches is substantial: a comprehensive systematic review and meta-analysis concluded that individuals adhering to healthiest lifestyles experienced a substantial reduction in all-cause mortality (55%), cardiovascular-specific mortality (58%), and the onset of CVD (62%). These benefits were consistent across various continents, ethnic groups, and socioeconomic strata, highlighting the universal advantage of concurrently addressing multiple lifestyle-related risk factors to alleviate the global burden of disease (16). Moreover, interventions including motivational interviewing have been proved to have a positive impact on different outcomes including tobacco abstinence, amount of alcohol, and marihuana use, when delivered across various medical settings, and a systematic review and meta-analysis showed durable effects after the intervention (24). However, a study on newly diagnosed hypertensive patients revealed that the main lifestyle adjustment after the diagnosis was smoking cessation, not necessarily precipitating other lifestyle changes, such alcohol reduction (13). Similarly, our research suggests that the period after a hospitalization for CVD can influence some lifestyle changes like smoking cessation while a substantial number of patients still require further interventions, particularly concerning alcohol use. This suggests that the challenges in ceasing alcohol consumption are persistent and have a more intricated nature, besides the experience of a cardiovascular event. The less pronounced reduction in alcohol consumption compared to tobacco use observed in our study may be explained by several interrelated factors. For example, a prevailing ambiguity surrounding the effects of moderate alcohol use on cardiovascular health contrast the unequivocal and longer recognized evidence on the risks of tobacco use. Previous research suggesting potential benefits of moderate alcohol use (25) may lead to a mixed public health message, compared to the clear forceful advisories against tobacco use. The normalization and high prevalence of alcohol use in our society could also contribute to the lack of consideration of it as a true cardiovascular risk factor. Other factors such as cultural context, specialized care, or social support could also influence strongly alcohol use patterns (26).

The absence of a significant effect of group in the prevalence and quantity of tobacco and alcohol use suggest that, while individuals may make positive changes in their substance use habits following a cardiovascular episode, these changes may not be sustained over time, potentially leading to relapse. This pattern may imply that, in the long term, there might be no significant distinctions between the groups regarding their use and the quantity of the substance intake. However, it is important to note that baseline differences were observed in the ASSIST scores for tobacco use. This finding raises the possibility that even if patients return to smoking after a cardiovascular event, the associated risks of their consumption might remain reduced compared to their pre-episode level. However, interventions should extend beyond the hospital setting to achieve long-term behaviour change, especially for alcohol use.

Several limitations must be acknowledged in the interpretation of the present study. The relatively small sample size of patients using tobacco and/or alcohol and the single-centre setting, may limit the interpretation and generalizability of the findings. Additionally, reliance in self-reported data for smoking and drinking habits, in those patients who were beyond the urinary detection window, may potentially compromise the accuracy of the data. However, self-report is recognized as a valid outcome measure in substance use disorder research by the EMA (27). Furthermore, the exclusion of patients with admission duration exceeding 15 days may have introduced selection bias by systematically excluding the most clinically complex patients with the highest comorbidity burden, who likely received more intensive and prolonged exposure to cessation interventions during hospitalization. This may limit generalizability and potentially underestimate the relationship between hospitalization and cessation outcomes among the highest-risk patients. Similarly, the exclusion of patients with language barriers may have introduced additional bias, as these patients may differ in health behaviors and access to cessation resources. Moreover, significant baseline differences between FE and CE groups (younger age, lower comorbidity burden, higher employment rates in FE) likely reflect underlying disease chronicity. While our models adjusted for age, CCI, psychiatric comorbidity, and ASSIST scores, employment status was not included, potentially introducing socioeconomic confounding. Furthermore, the absence of biomarker validation during follow-up telephone interviews, might have resulted in an underreporting of the prevalence of tobacco and alcohol use. The attrition of participants and the subsequent loss of information during follow-up may have also impacted the results and introduced potential bias. Our study did not evaluate predictors of more substantial changes in tobacco and alcohol use behaviours. However, some strengths shouldn't be overlooked: the naturalistic, longitudinal design of the study, the careful, systematic, and real-time assessment of the tobacco and alcohol use using validated methods, and the statistical analysis of the group's trajectories. Future research should aim to identify patients who may face greater challenges in implementing lifestyle modifications related to substance use. Identifying these individuals would enable the provision of more effective and tailored interventions.

### 4.1 Conclusions

Cardiovascular hospitalization represents a critical window for behavioral intervention, with patients demonstrating substantial tobacco reduction but more modest changes in alcohol consumption. Healthcare providers should adopt an approach that encompasses the concurrent measurement of alcohol and tobacco exposure, and the implementation of integrated brief interventions, advocating for moderation or, optimally, cessation, to align with the best practices for cardiovascular health. Approaches such as motivational interviewing may facilitate and sustain behavioural changes in tobacco and alcohol use.

### 4.2 Implications

This study highlights:

- Hospitalization for cardiovascular events offers a critical opportunity to influence substance use behaviors, particularly smoking cessation.
- Alcohol consumption tends to persist despite interventions, and there are no significant group-by-time interactions observed.
- Patients who continue smoking during consecutive hospital admissions (rather than at their first cardiovascular event) should undergo thorough assessment, as tailored interventions are essential. Smoking reduction appears less evident in these cases compared to first-time CVD episodes.
- This highlights the need for strategies that address both tobacco and alcohol use, as alcohol is often underestimated as a cardiovascular risk factor.
- Implementing such measures could improve the effectiveness of behavioral change efforts and reduce relapse risk.
- Ultimately, these interventions can lead to better long-term cardiovascular outcomes.

## Data Availability

The raw data supporting the conclusions of this article will be made available by the authors, without undue reservation.
